# Epidemiological characteristics of 1385 primary sacral tumors in one institution in China

**DOI:** 10.1186/s12957-020-02045-w

**Published:** 2020-11-12

**Authors:** Jun Wang, Dasen Li, Rongli Yang, Xiaodong Tang, Taiqiang Yan, Wei Guo

**Affiliations:** Musculoskeletal Tumor Center, Peking University People’s Hospital, No. 11 Xizhimen South Street, Beijing, 100044 China

**Keywords:** Primary sacral tumors, Epidemiology, Sacrum, Bone tumor

## Abstract

**Background:**

Sacral tumors and tumor-like lesions are a rare group of lesions that can affect children and adults of all ages. Little is known about clinical characteristics of age, gender, histologic type, and anatomic site in China.

**Methods:**

A total of 1385 patients with sacral tumors and tumor-like lesions, which had the clinical record at our bone tumor center from January 2000 to November 2018 were analyzed. The metastatic cancers were not included in the present study.

**Results:**

A total of 51.7% (716 cases) were malignant and 48.3% (669 cases) were benign tumors or tumor-like lesions. Of malignant tumors, chordoma was the most common malignant tumor (316 cases, 22.8% of all tumors), followed by chondrosarcoma, myeloma, and other histologic types. The most common histological type of benign tumors was a giant cell tumor accounting for 14.8% (205 cases) of all tumors, followed by neurofibroma, schwannoma, and other types. The most common age group affected by malignant bone tumors was the 51- to 60-year-old group, followed by the 41- to 50-year-old group. The most commonly affected age group for benign tumors and tumor-like lesions was the 31- to 50-year-old group, followed by the 21- to 30-year old group. Furthermore, the following histologic types had gender predilection. Chordoma, chondrosarcoma, myeloma, and osteosarcoma affected more frequently males than females. Malignant peripheral nerve sheath tumor, lymphoma, giant cell tumor, neurofibroma, tuberculosis, teratoma, and epidermoid cyst more frequently affected females than males.

**Conclusions:**

The large cohort of sacral tumors and tumor-like lesions in our database may reveal their clinical characteristics of age, gender, histologic type, and anatomic site in China and features of sacral tumors and tumor-like lesions are fairly distinct from the mobile spine and extremities.

## Introduction

Sacral tumors and tumor-like lesions are a rare group of tumors that can affect children and adults of all ages. Diagnosing a sacral tumor at an early stage is a great challenge because of the lack of specific clinical symptoms and sacral tumors are often extensively involved in the sacral nerves, iliac vessels, and other surrounding organs. Furthermore, the management could be quite difficult for orthopedic surgeons because of its complicated anatomy and high risk of postoperative complications. The surgical treatment of these tumors has more difficult because of the complex regional anatomy. Surgeons must not only be familiar with local anatomy from a neurologic, colorectal, urologic, gynecological, orthopedic, and plastic standpoint but also sometimes have to face the dilemma between functional preservation and cure of the tumor [1–4].

A great number of specific histologic subtypes of them have been delineated, each of them with a unique appearance and biological potential [1, 2]. A barrier is presented to etiologic study due to the rarity and heterogeneity and it poses a great challenge in the understanding of clinical features [3]. Little is known about their clinical features of age, gender, histologic type, and anatomic site in China. China is a relatively big country with a steady population and the number of patients with sacral tumors and tumor-like lesions is huge and our hospital is the biggest specialized musculoskeletal tumor cancer in China. This provides us a beneficial condition to study clinical characteristics of sacral tumors and tumor-like lesions. The present study is aiming to provide the first analysis of features of age, gender, histologic type, and anatomic site for sacral tumors and tumor-like lesions not including metastatic cancers in our institution between January 2000 and November 2018.

## Patients and methods

We retrospectively reviewed 1385 patients with sacral tumors and tumor-like lesions, which had the clinical record at our bone tumor center from January 2000 to November 2018. There were 709 male and 676 female patients with a mean age of 43.9 ± 17.0 years (range, 2–86 years). Histologic diagnosis was confirmed by biopsy or operative specimen. The inclusion criteria for the present study were as follows: (1) patients had the definitive histological diagnosis; (2) diagnostic time was from the year 2000 to 2018. The exclusion criteria were as follows: (1) without a confirmed histological diagnosis; (2) diagnostic time was beyond the range of 2000 to 2018; (3) metastatic cancers at the sacrum. All patients which were included in the present study were given written informed consent for their data to be included in this study during the follow-up. All data were obtained from the clinical and radiograph records. This study was approved by the Institutional Review Board/Ethics Committee of the authors’ institution. The following data were collected in the present study: age, gender, affected sacrum level, pathological diagnosis. For the analysis of tumor location distribution, Region S_1_ or S_2_ or S_1–2_ can be described as a high level of sacrum and Region S_3_ or below S_3_ as a low level of sacrum.

For the initial clinical evaluation and diagnosis, all patients received plain radiographs, CT, MRI, and bone scanning. After imaging, we performed the needle biopsy to clarify the diagnosis. For the malignant tumors, the routine follow-up including clinical examination, radiographs of the extremity and chest were performed every 3 months for the first 6 months, every 6 months for the first 3 years, and then annually. The chest CT scanning was performed every 6 months for the first 3 years, and then annually. For the benign tumors, the routine follow-up was performed every 6 months for the first year and then annually.

### Statistical analysis

Continuous variables were summarized with means and ranges; categorical variables were summarized with frequency counts and percentages. The Student’s *t* test was used to compare the age in different histologic types of male and female in Tables 5 and 6. The chi-square test was used to compare the location distribution in Table 7. The SPSS software (version 19.0; SPSS Inc, Chicago, IL, USA) was used for all statistical analyses. *P* ≦ 0.05 indicated a statistically significant difference.

## Results

### Incidence of histological subtypes

**Fig. 1:**
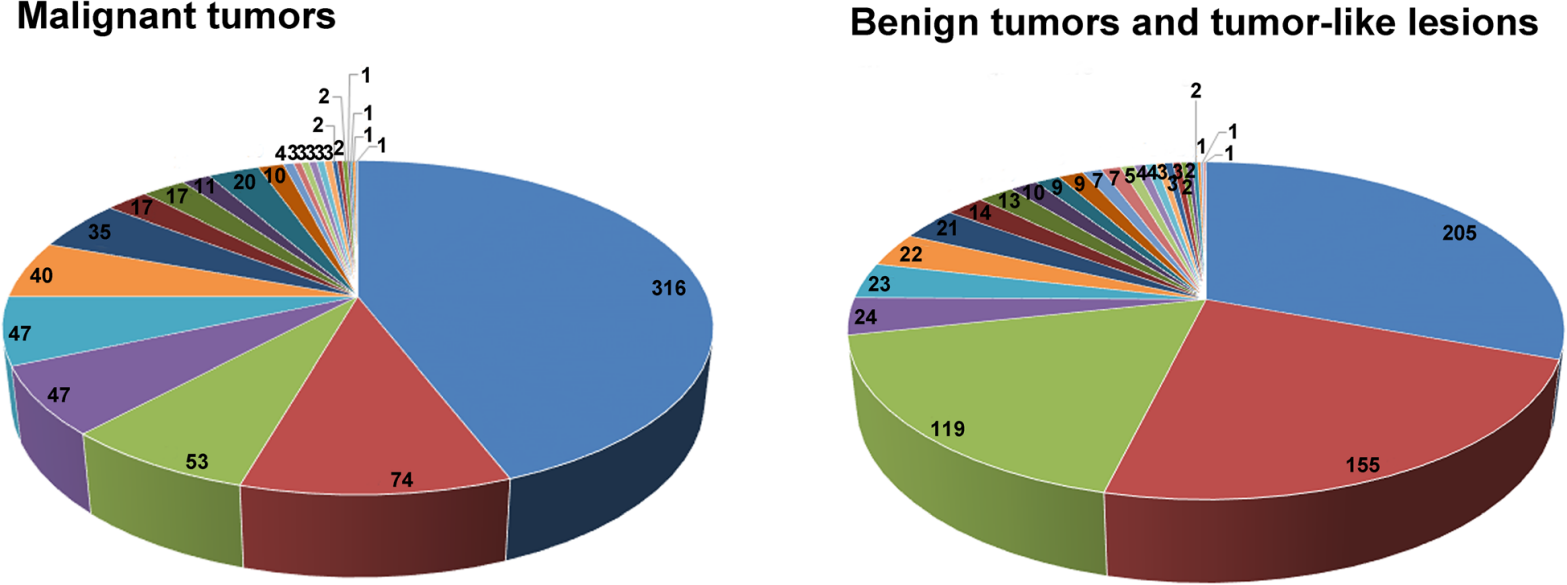
**Malignant tumors** Chordoma (316, 44.1%); Chondrosarcoma (74, 10.3%); Multiple myeloma (53, 7.4%); Malignant peripheral nerve sheath tumor (47, 6.6%); Ewing sarcoma (47, 6.6%); Osteosarcoma (40, 5.6%); Lymphoma (35, 4.9%); Solitary fibrous tumor (20, 2.8%); Spindle cell undifferentiated sarcoma (17, 2.4%); Malignant giant cell tumor (17, 2.4%); Malignant teratoma (11, 1.5%); Liposarcoma (10, 1.4%); Ependymoma (4, 0.6%); Angiosarcoma (3, 0.4%); Fibrosarcoma (3, 0.4%); Hemangioendothelima (3, 0.4%); Myelocytic sarcoma (3, 0.4%); Yolk sac tumor (3, 0.4%); Leiomyosarcoma (2, 0.3%); Alveolar soft part sarcoma (2, 0.3%); Leukemia (2, 0.3%); Epithelioid sarcoma (1, 0.1%); Granulocyte sarcoma (1, 0.1%); Myofibroblastic sarcoma (1, 0.1%); Synovial sarcoma (1, 0.1%) **Benign tumors and tumor-like lesions** Giant cell tumor (205, 30.6%); Neurofibroma (155, 23.2%); Schwannoma (119, 17.8%); Tuberculosis (24, 3.6%); Teratoma (23, 3.4%); Sacral canal cysts (22, 3.3%); Epidermoid cyst (21, 3.1%); Hemangioma (14, 2.1%); Meningeal cysts (13, 1.9%); Primary aneurysmal bone cyst (10, 1.5%); Fibrous dysplasia (9, 1.3%); Osteoblastoma (9, 1.3%); Eosinophilic granuloma (7, 1.0%); Simple bone cyst (7, 1.0%); Spinal meningioma (5, 0.7%); Gut-tail cyst (4, 0.6%); Benign fibrous histiocytoma (4, 0.6%); Fibromatosis (3, 0.4%); Diffuse giant cell tumor of tendon sheath (3, 0.4%); Chondroblastoma (3, 0.4%); Lipoma (2, 0.3%); Phosphouria stromal tumor (2, 0.3%); Osteoidosteoma (2, 0.3%); Liomyoma (1, 0.1%); Paget disease (1, 0.1%); Osteochondroma (1, 0.1%)

**Fig. 2:**
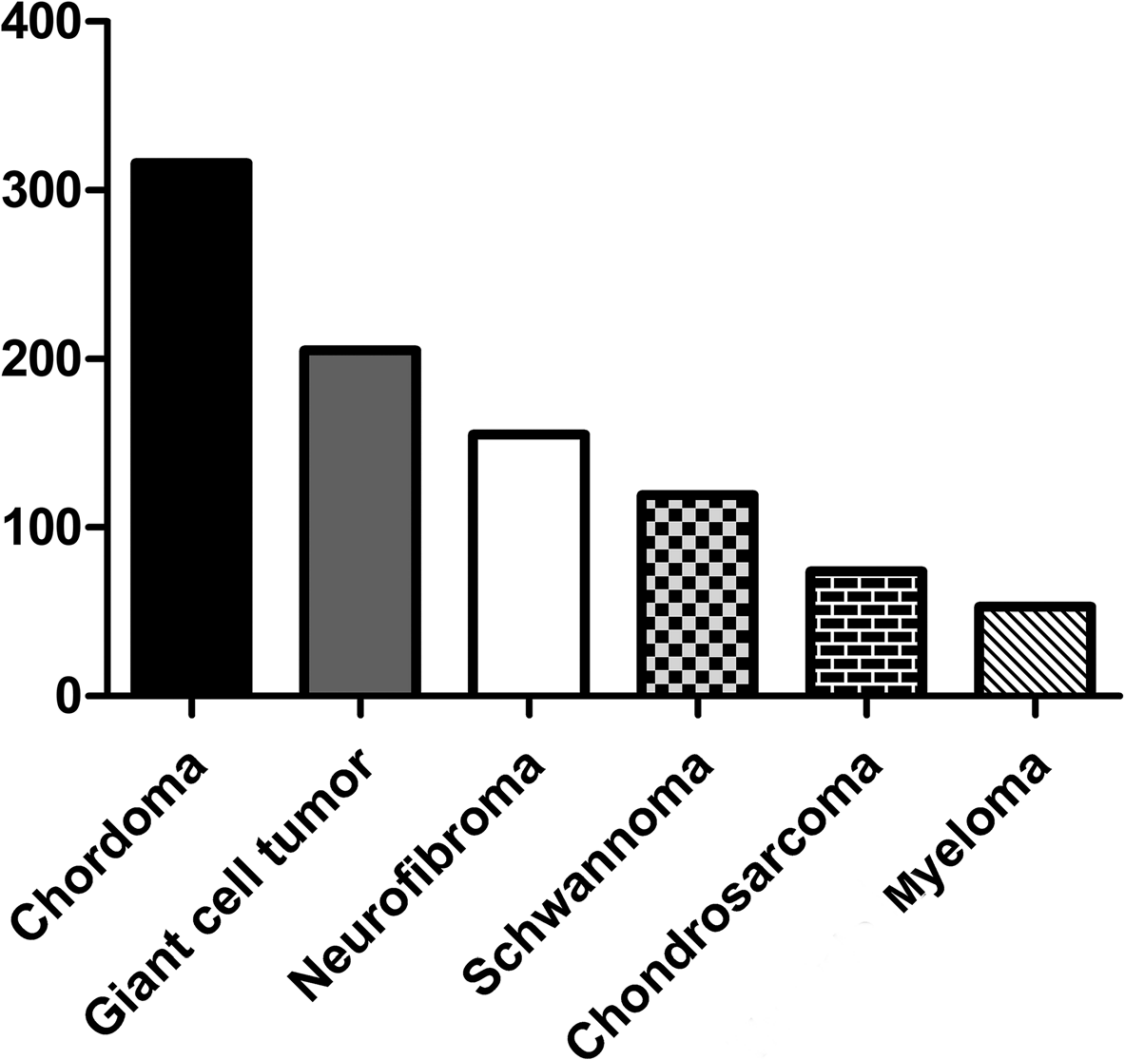
Top six histological types of primary sacral tumors

The histological types of sacral tumors and tumor-like lesions were listed in Tables 1 and 2. A total of 51.7% (716 cases) were malignant and 48.3% (669 cases) were benign tumors and tumor-like lesions (Fig. 1). Of malignant tumors, chordoma was the most common malignant type of PST (22.8% of all PST, 316 cases), followed by chondrosarcoma (5.3%, 74 cases), myeloma (3.8%, 53 cases), and malignant peripheral nerve sheath tumor (3.4%, 47 cases) (Table 1). The most common histological type of benign tumors and tumor-like lesions was giant cell tumor accounting for 14.8% (205 cases), followed by neurofibroma (11.2%, 155 cases), schwannoma (8.6%, 119 cases), and tuberculosis (1.7%, 24 cases) (Table 2). The top six of primary sacral tumors were summarized in Fig. 2.

**Table 1:** Histopathological diagnosis of 716 patients with sacral malignant tumors

Primary malignant tumor	No.(percentage) in malignant tumors	No.(percentage) in all patients
Chordoma	316 (44.1%)	316 (22.8%)
Chondrosarcoma	74 (10.3%)	74 (5.3%)
Myeloma	53 (7.4%)	53 (3.8%)
Malignant peripheral nerve sheath tumor	47 (6.6%)	47 (3.4%)
Ewing sarcoma	47 (6.6%)	47 (3.4%)
Osteosarcoma	40 (5.6%)	40 (2.9%)
Lymphoma	35 (4.9%)	35 (2.5%)
Solitary fibrous tumor	20 (2.8%)	20 (1.4%)
Undifferentiated pleomorphic sarcoma	17 (2.4%)	17 (1.2%)
Malignant giant cell tumor	17 (2.4%)	17 (1.2%)
Malignant teratoma	11 (1.5%)	11 (0.8%)
Liposarcoma	10 (1.4%)	10 (0.7%)
Ependymoma	4 (0.6%)	4 (0.3%)
Angiosarcoma	3 (0.4%)	3 (0.2%)
Fibrosarcoma	3 (0.4%)	3 (0.2%)
Hemangioendothelima	3 (0.4%)	3 (0.2%)
Myelocytic sarcoma	3 (0.4%)	3 (0.2%)
Yolk sac tumor	3 (0.4%)	3 (0.2%)
Leiomyosarcoma	2 (0.3%)	2 (0.1%)
Alveolar soft part sarcoma	2 (0.3%)	2 (0.1%)
Leukemia	2 (0.3%)	2 (0.1%)
Epithelioid sarcoma	1 (0.1%)	1 (0.07%)
Granulocyte sarcoma	1 (0.1%)	1 (0.07%)
Myofibroblastic sarcoma	1 (0.1%)	1 (0.07%)
Synovial sarcoma	1 (0.1%)	1 (0.07%)

**Table 2:** Histopathological diagnosis of 669 patients with sacral benign tumors and tumor-like lesions

Primary benign tumor	No.(percentage) in BT/TLL	No.(percentage) in all patients
Giant cell tumor	205 (30.6%)	205 (14.8%)
Neurofibroma	155 (23.2%)	155 (11.2%)
Schwannoma	119 (17.8%)	119 (8.6%)
Tuberculosis	24 (3.6%)	24 (1.7%)
Teratoma	23 (3.4%)	23 (1.7%)
Sacral canal cysts	22 (3.3%)	22 (1.6%)
Epidermoid cyst	21 (3.1%)	21 (1.5%)
Hemangioma	14 (2.1%)	14 (1.0%)
Meningeal cysts	13 (1.9%)	13 (0.9%)
Primary aneurysmal bone cyst	12 (1.8%)	12 (0.9%)
Fibrous dysplasia	9 (1.3%)	9 (0.6%)
Osteoblastoma	9 (1.3%)	9 (0.6%)
Eosinophilic granuloma	7 (1.0%)	7 (0.5%)
Simple bone cyst	5 (0.7%)	5 (0.4%)
Spinal meningioma	5 (0.7%)	5 (0.4%)
Gut-tail cyst	4 (0.6%)	4 (0.3%)
Benign fibrous histiocytoma	4 (0.6%)	4 (0.3%)
Fibromatosis	3 (0.4%)	3 (0.2%)
Diffuse giant cell tumor of tendon sheath	3 (0.4%)	3 (0.2%)
Chondroblastoma	3 (0.4%)	3 (0.2%)
Lipoma	2 (0.3%)	2 (0.1%)
Phosphouria stromal tumor	2 (0.3%)	2 (0.1%)
Osteoidosteoma	2 (0.3%)	2 (0.1%)
Leiomyoma	1 (0.1%)	1 (0.07%)
Paget disease	1 (0.1%)	1 (0.07%)
Osteochondroma	1 (0.1%)	1 (0.07%)

*BT/TLL* benign tumor and tumor-like lesions

### Age distribution of sacral tumors and tumor-like lesions

For a total of 1385 sacral tumors and tumor-like lesions, the mean age was 43.9 ± 17.0 years (range, 2–86 years), 51.2% (709 cases) of the lesions occurred in males and 48.8% (676 cases) in females, with a mean age of 44.5 ± 17.2 and 43.1 ± 16.7 years. The mean age of 716 malignant and 669 benign tumors and tumor-like lesions groups were respectively 46.9 ± 17.5 years (range, 2–86 years) and 40.6 ± 15.7 years (range, 2–85 years).

The incidences were presented separately by gender (males and females) and age at diagnosis was grouped into nine subgroups (from 0 to 10 years cohort to ≥80 years cohort). The most common age group affected by malignant bone tumors was the 51- to 60-year-old group (21.1%, 151cases), followed by the 41- to 50-year-old group (19.4%, 139 cases) (Table 3). The most commonly affected age group for benign tumors and tumor-like lesions were the 31- to 40-year-old and 41- to 50-year-old groups (21.1%, 141 cases and 21.1%, 141 cases), followed by the 21- to 30-year old group (19.6%, 131 cases) (Table 4).

**Table 3:** Age distribution of sacral malignant tumors

Histology	0–10	11–20	21–30	31–40	41–50	51–60	61–70	71–80	81–90	Total
**Malignant tumors**	9(1.3%)	54(7.5%)	88(12.3%)	96(13.4%)	139(19.4%)	151(21.1%)	122(17.0%)	52(7.3%)	5(0.7%)	716
Chordoma	0(0)	1(0.3%)	17(5.4%)	29(9.2%)	58(18.4%)	85(26.9%)	82(25.9%)	39(12.3%)	5(1.6%)	316
Chondrosarcoma	0(0)	5(6.8%)	14(18.9%)	16(21.6%)	19(25.7%)	16(21.6%)	4(5.4%)	0(0)	0(0)	74
Myeloma	0(0)	0(0)	2(3.8%)	7(13.2%)	13(24.5%)	13(24.5%)	12(22.6%)	6(11.3%)	0(0)	53
Malignant peripheral nerve sheath tumor	1(2.1%)	5(10.6%)	10(21.3%)	5(10.6%)	11(23.4%)	9(19.1%)	5(10.6%)	1(2.1%)	0(0)	47
Ewing sarcoma	5(10.6%)	19(40.4%)	9(19.1%)	9(19.1%)	4(8.5%)	1(2.1%)	0(0)	0(0)	0(0)	47
Osteosarcoma	1(2.5%)	15(37.5%)	13(32.5%)	5(12.5%)	3(7.5%)	3(7.5%)	0(0)	0(0)	0(0)	40
Lymphoma	0(0)	2(5.7%)	4(11.4%)	5(14.3%)	9(25.7%)	9(25.7%)	4(11.4%)	2(5.7%)	0(0)	35
Solitary fibrous tumor	0(0)	0(0)	4(20%)	5(25%)	4(20%)	2(10%)	4(20%)	1(5%)	0(0)	20
Undifferentiated pleomorphic sarcoma	0(0)	0(0)	1(5.9%)	2(11.8%)	3(17.6%)	5(29.4%)	4(23.5%)	2(11.8%)	0(0)	17
Malignant giant cell tumor	0(0)	2(11.8%)	6(35.3%)	4(23.5%)	2(11.8%)	2(11.8%)	1(5.9%)	0(0)	0(0)	17
Malignant teratoma	0(0)	0(0)	2(18.2%)	4(36.4%)	1(9.1%)	2(18.2%)	2(18.2%)	0(0)	0(0)	11
Liposarcoma	0(0)	0(0)	0(0)	2(20.0%)	4(40.0%)	1(10.0%)	3(30.0%)	0(0)	0(0)	10
Ependymoma	0(0)	0(0)	1(25.0%)	0(0)	2(50.0%)	0(0)	0(0)	1(25.0%)	0(0)	4
Angiosarcoma	0(0)	0(0)	0(0)	0(0)	1(33.3%)	2(67.7%)	0(0)	0(0)	0(0)	3
Fibrosarcoma	0(0)	1(33.3%)	0(0)	0(0)	2(67.7%)	0(0)	0(0)	0(0)	0(0)	3
Hemangioendothelima	1(33.3%)	0(0)	0(0)	1(33.3%)	0(0)	0(0)	1(33.3%)	0(0)	0(0)	3
Myelocytic sarcoma	0(0)	0(0)	1(33.3%)	2(67.7%)	0(0)	0(0)	0(0)	0(0)	0(0)	3
Yolk sac tumor	1(33.3%)	2(67.7%)	0(0)	0(0)	0(0)	0(0)	0(0)	0(0)	0(0)	3
Leiomyosarcoma	0(0)	0(0)	1(50.0%)	0(0)	1(50.0%)	0(0)	0(0)	0(0)	0(0)	2
Alveolar soft part sarcoma	0(0)	0(0)	2(100.0%)	0(0)	0(0)	0(0)	0(0)	0(0)	0(0)	2
Leukemia	0(0)	1(50.0%)	0(0)	0(0)	1(50.0%)	0(0)	0(0)	0(0)	0(0)	2
Epithelioid sarcoma	0(0)	1(100.0%)	0(0)	0(0)	0(0)	0(0)	0(0)	0(0)	0(0)	1
Granulocyte sarcoma	0(0)	0(0)	0(0)	0(0)	1(100.0%)	0(0)	0(0)	0(0)	0(0)	1
Myofibroblastic sarcoma	0(0)	0(0)	0(0)	0(0)	0(0)	1(100.0%)	0(0)	0(0)	0(0)	1
Synovial sarcoma	0(0)	0(0)	1(100.0%)	0(0)	0(0)	0(0)	0(0)	0(0)	0(0)	1

**Table 4:** Age distribution of sacral benign tumors and tumor-like lesions

Histology	0–10	11–20	21–30	31–40	41–50	51–60	61–70	71–80	81–90	Total
Primary benign tumor	11(1.6%)	58(8.7%)	131(19.6%)	141(21.1%)	141(21.1%)	108(16.1%)	60(9.0%)	16(2.4%)	3(0.4%)	669
Giant cell tumor	0(0)	24(11.7%)	61(29.8%)	62(30.2%)	40(19.5%)	12(5.9%)	6(2.9%)	0(0)	0(0)	205
Neurofibroma	1(0.6%)	6(3.9%)	18(11.6%)	24(15.5%)	39(25.2%)	41(26.5%)	20(12.9%)	5(3.2%)	1(0.6%)	155
Schwannoma	0(0)	2(1.7%)	16(13.4%)	24(20.2%)	33(27.7%)	24(20.2%)	16(13.4%)	4(3.4%)	0(0)	119
Tuberculosis	1(4.2%)	2(8.3%)	4(16.7%)	5(20.8%)	4(16.7%)	4(16.7%)	1(4.2%)	3(12.5%)	0(0)	24
Teratoma	1(4.3%)	3(13.0%)	8(34.8%)	6(26.1%)	3(13.0%)	1(4.3%)	1(4.3%)	0(0)	0(0)	23
Sacral canal cysts	0(0)	1(4.5%)	3(13.6%)	4(18.2%)	3(13.6%)	4(18.2%)	6(27.3%)	0(0)	1(4.5%)	22
Epidermoid cyst	0(0)	2(9.5%)	3(14.3%)	2(9.5%)	5(23.8%)	4(19.0%)	2(9.5%)	3(14.3%)	0(0)	21
Hemangioma	1(7.1%)	2(14.3%)	1(7.1%)	2(14.3%)	1(7.1%)	4(28.6%)	1(7.1%)	1(7.1%)	1(7.1%)	14
Meningeal cysts	0(0)	1(7.7%)	1(7.7%)	1(7.7%)	1(7.7%)	6(46.2%)	3(23.1%)	0(0)	0(0)	13
Primary aneurysmal bone cyst	2(16.7%)	8(66.7%)	1(8.3%)	0(0)	1(8.3%)	0(0)	0(0)	0(0)	0(0)	12
Fibrous dysplasia	0(0)	1(11.1%)	2(22.2%)	3(33.3%)	2(22.2%)	1(11.1%)	0(0)	0(0)	0(0)	9
Osteoblastoma	3(33.3%)	3(33.3%)	1(11.1%)	0(0)	1(11.1%)	1(11.1%)	0(0)	0(0)	0(0)	9
Eosinophilic granuloma	1(14.3%)	1(14.3%)	1(14.3%)	2(28.6%)	0(0)	2(28.6%)	0(0)	0(0)	0(0)	7
Simple bone cyst	0(0)	0(0)	1(20.0%)	1(20.0%)	1(20.0%)	2(40.0%)	0(0)	0(0)	0(0)	5
Spinal meningioma	0(0)	0(0)	1(20.0%)	2(40.0%)	2(40.0%)	0(0)	0(0)	0(0)	0(0)	5
Gut-tail cyst	0(0)	0(0)	3(75.0%)	0(0)	1(25.0%)	0(0)	0(0)	0(0)	0(0)	4
Benign fibrous histiocytoma	0(0)	0(0)	1(25.0%)	1(25.0%)	1(25.0%)	0(0)	1(25.0%)	0(0)	0(0)	4
Fibromatosis	0(0)	0(0)	0(0)	0(0)	2(66.7%)	1(33.3%)	0(0)	0(0)	0(0)	3
Diffuse giant cell tumor of tendon sheath	0(0)	1(33.3%)	1(33.3%)	1(33.3%)	0(0)	0(0)	0(0)	0(0)	0(0)	3
Chondroblastoma	1(33.3%)	0(0)	2(66.7%)	0(0)	0(0)	0(0)	0(0)	0(0)	0(0)	3
Lipoma	0(0)	0(0)	0(0)	0(0)	0(0)	1(50.0%)	1(50.0%)	0(0)	0(0)	2
Phosphouria stromal tumor	0(0)	0(0)	1(50.0%)	0(0)	0(0)	0(0)	1(50.0%)	0(0)	0(0)	2
Osteoidosteoma	0(0)	1(50.0%)	1(50.0%)	0(0)	0(0)	0(0)	0(0)	0(0)	0(0)	2
Leiomyoma	0(0)	0(0)	0(0)	0(0)	1(100.0%)	0(0)	0(0)	0(0)	0(0)	1
Paget disease	0(0)	0(0)	0(0)	0(0)	0(0)	0(0)	1(100.0%)	0(0)	0(0)	1
Osteochondroma	0(0)	0(0)	0(0)	1(100.0%)	0(0)	0(0)	0(0)	0(0)	0(0)	1

**Fig. 3:**
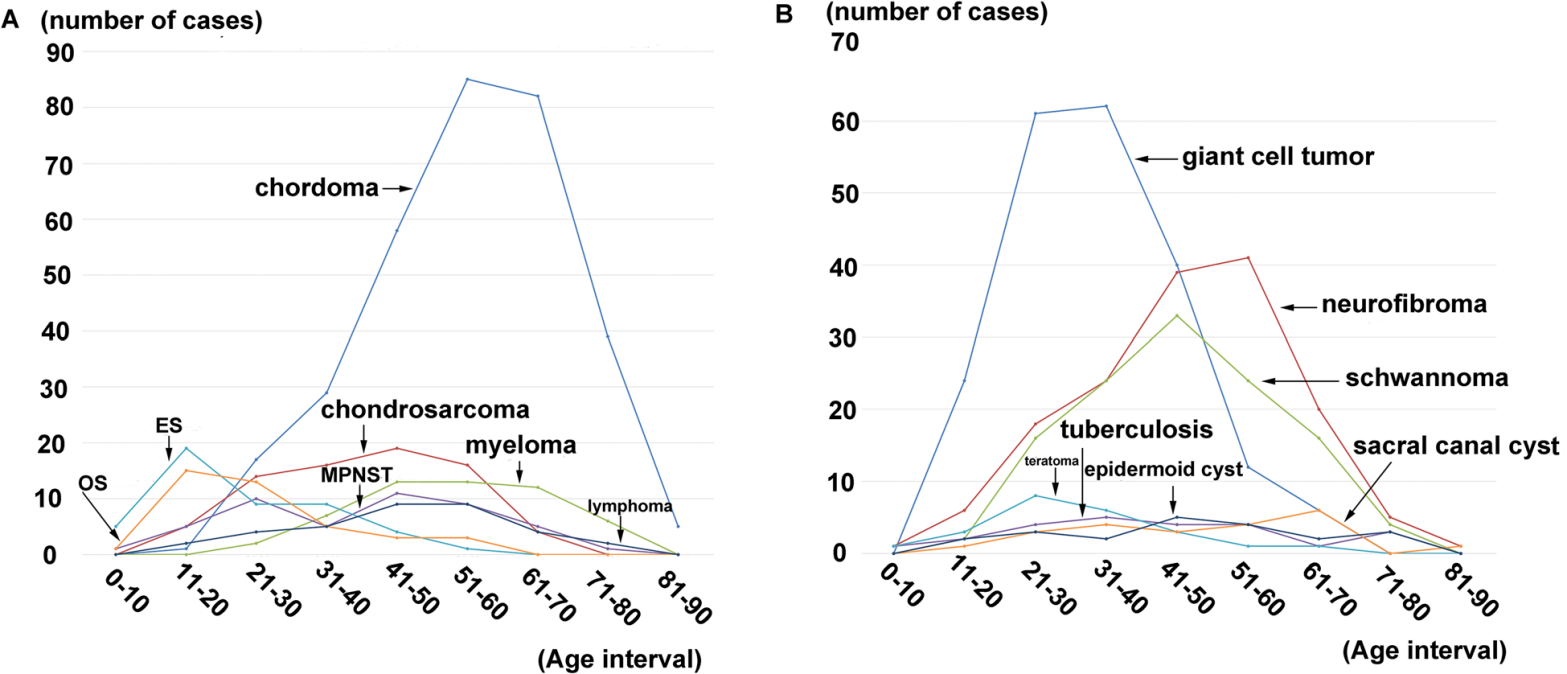
**a–b** Clinical features of gender for top seven of primary sacral malignant tumors; **c–d** Clinical features of gender for top seven of primary sacral benign tumors

We analyzed the age and gender-related clinical characteristics of the top seven primary sacral malignant tumors (Fig. 3). Of 316 chordomas, the mean age was 55.4 ± 13.7 (range, 18–86 years) and 210 cases occurred in males and 106 cases in females, with a mean age of 56.1 ± 13.3 and 54.1 ± 14.4 years. Of 74 chondrosarcoma, the mean age was 40.9 ± 13.3 (range, 17–69 years) and 41 cases occurred in males and 33 cases in females, with a mean age of 37.7 ± 14.8 and 44.9 ± 10.0 years. Of 53 myeloma, the mean age was 54.1 ± 13.2 (range, 22–78 years) and 41 cases occurred in males and 33 cases in females, with a mean age of 53.4 ± 14.4 and 55.6 ± 10.6 years. Of 47 malignant peripheral nerve sheath tumors, the mean age was 40.8 ± 16.7 (range, 8–75 years) and 19 cases occurred in males and 28 cases in females, with a mean age of 40.4 ± 18.3 and 41.0 ± 15.8 years (Table 5). Moreover, we analyzed the age and gender-related clinical characteristics of the top seven primary sacral benign tumors and tumor-like lesions (Fig. 3). Of 205 giant cell tumors, the mean age was 34.4 ± 11.6 (range, 11–67 years) and 69 cases occurred in males and 86 cases in females, with a mean age of 32.7 ± 10.3 and 35.8 ± 12.5 years. Of 155 neurofibroma, the mean age was 46.7 ± 14.5 (range, 9–83 years) and 95 cases occurred in males and 110 cases in females, with a mean age of 44.6 ± 13.8 and 48.4 ± 14.9 years. Of 119 schwannoma, the mean age was 46.1 ± 13.5 (range, 13–79 years) and 60 cases occurred in males and 59 cases in females, with a mean age of 44.6 ± 13.1 and 47.7 ± 13.9 years (Table 6).

**Table 5:** Gender distribution of sacral malignant tumors

Histology	No.	Male		Female		M:F	Age range (years)	Mean ± SD (age)		
	No.	No.	%	No.	%			Male	Female	Total *P* value
Chordoma	316	210	66.5%	106	33.5%	1.98:1	18–86	56.1 ± 13.3	54.1 ± 14.4	55.4 ± 13.7 *P* = 0.236
Chondrosarcoma	74	41	55.4%	33	44.6%	1.24:1	17–69	37.7 ± 14.8	44.9 ± 10.0	40.9 ± 13.3 *P* = 0.015
Myeloma	53	36	67.9%	17	32.1%	2.12:1	22–78	53.4 ± 14.4	55.6 ± 10.6	54.1 ± 13.2 *P* = 0.567
Malignant peripheral nerve sheath tumor	47	19	40.4%	28	59.6%	0.68:1	8–75	40.4 ± 18.3	41.0 ± 15.8	40.8 ± 16.7 *P* = 0.903
Ewing sarcoma	47	24	51.1%	23	48.9%	1.04:1	2–56	23.8 ± 13.1	22.7 ± 12.8	23.3 ± 12.8 *P* = 0.791
Osteosarcoma	40	23	57.5%	17	42.5%	1.35:1	10–58	29.0 ± 13.6	22.0 ± 10.3	26.0 ± 12.7 *P* = 0.085
Lymphoma	35	15	42.9%	20	57.1%	0.75:1	20–78	46.2 ± 16.4	49.2 ± 14.3	47.9 ± 15.0 *P* = 0.574

**Table 6:** Gender distribution of sacral benign tumors and tumor-like lesions

Histology	No.	Male		Female		M:F	Age range (years)	Mean ± SD (age)		
	No.	No.	%	No.	%			Male	Female	Total *P* value
Giant cell tumor	205	95	46.3%	110	53.7%	0.86:1	11–67	32.7 ± 10.3	35.8 ± 12.5	34.4 ± 11.6 *P* = 0.057
Neurofibroma	155	69	44.5%	86	55.5%	0.80:1	9–83	44.6 ± 13.8	48.4 ± 14.9	46.7 ± 14.5 *P* = 0.108
Schwannoma	119	60	50.4%	59	49.6%	1.02:1	13–79	44.6 ± 13.1	47.7 ± 13.9	46.1 ± 13.5 *P* = 0.212
Tuberculosis	24	8	33.3%	16	66.7%	0.50:1	8–76	46.9 ± 21.6	39.5 ± 17.7	42.0 ± 18.9 *P* = 0.380
Teratoma	23	4	17.4%	19	82.6%	0.21:1	5–62	33.8 ± 17.3	31.3 ± 12.4	31.7 ± 12.9 *P* = 0.741
Sacral canal cysts	22	11	50.0%	11	50.0%	1.00:1	19–81	46.0 ± 21.2	51.5 ± 12.3	48.8 ± 17.1 *P* = 0.461
Epidermoid cyst	21	4	19.0%	17	81.0%	0.24:1	19–78	32.8 ± 23.0	50.3 ± 16.4	47.0 ± 18.6 *P* = 0.089

### Gender characteristics of sacral tumors and tumor-like lesions

**Fig. 4:**
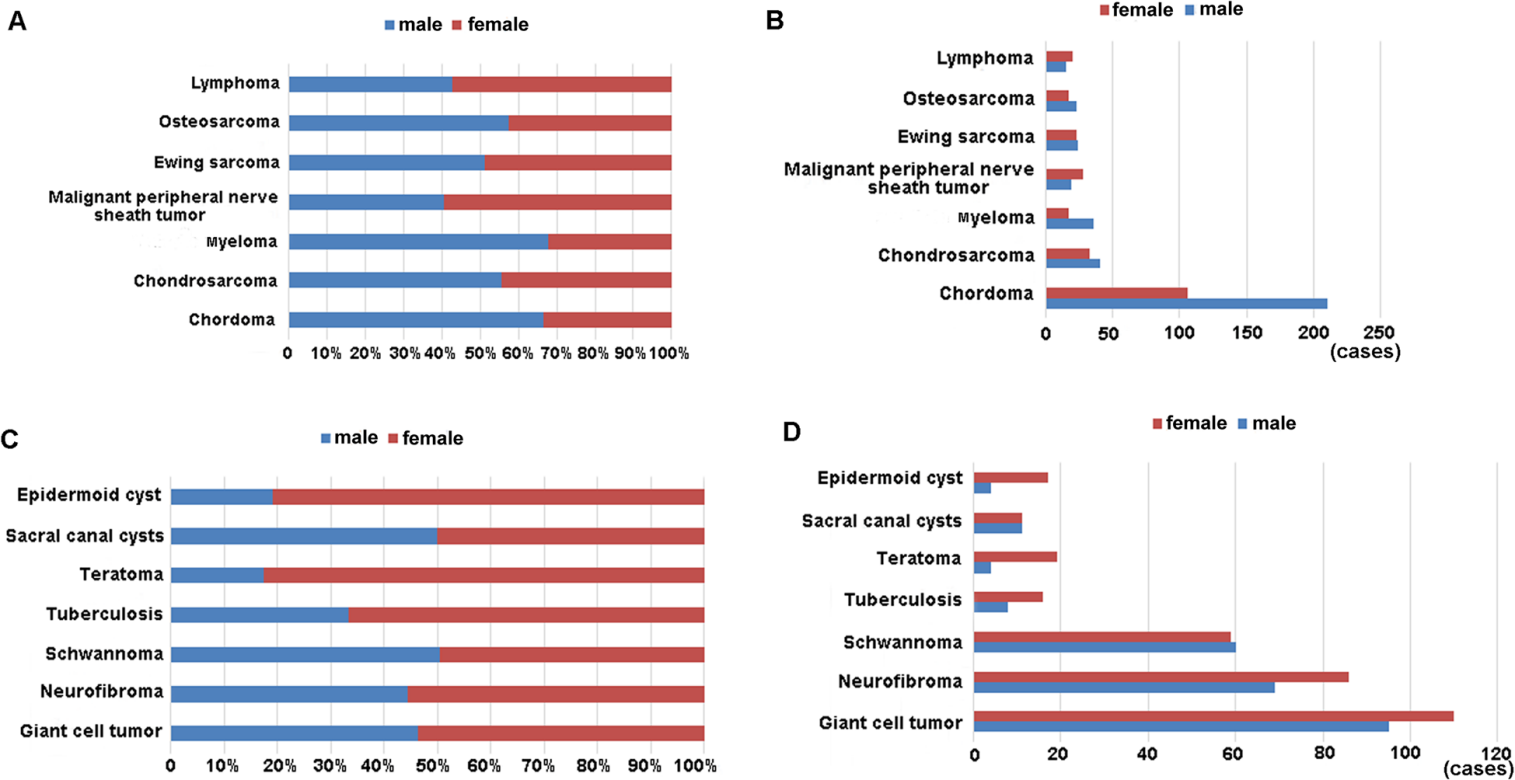
**a** Age distribution for top seven of primary sacral malignant tumors. *N* = numbers of patients; **b** Age distribution for top seven of primary sacral benign tumors. *N* = numbers of patients

The distribution of different histological types in males and females was shown as follows (Fig. 4): chordoma (316 cases, M: F = 1.98:1), chondrosarcoma (74 cases, M: F = 1.24:1), myeloma (53 cases, M: F = 2.12:1), and osteosarcoma (40 cases, M: F = 1.35:1) affected more frequently males than females. Meanwhile, malignant peripheral nerve sheath tumor (47 cases, M: F = 0.68:1), lymphoma (35 cases, M: F = 0.75:1), giant cell tumor (205 cases, M: F = 0.86:1), neurofibroma (155 cases, M: F = 0.80:1), tuberculosis (24 cases, M: F = 0.50:1), teratoma (23 cases, M: F = 0.21:1) and epidermoid cyst (21 cases, M: F = 0.24:1) affected more frequently females than males (Tables 5 and 6).

### Location distribution of sacral tumors and tumor-like lesions

**Fig. 5:**
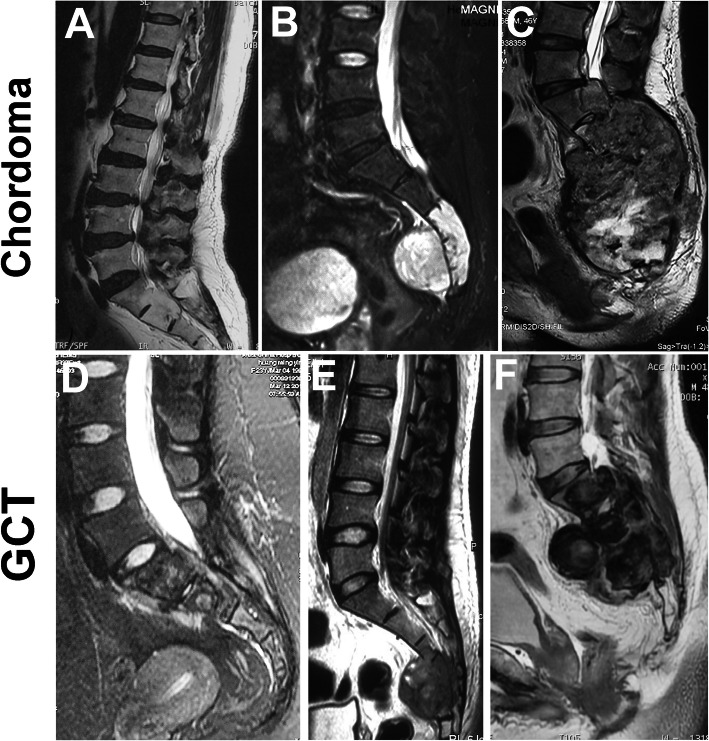
Location distribution of chordoma and giant cell tumor. **a** Chordomas at the Region S_1_ or S_2_ or S_1-2_ accounting for 1.6% (5/316); **b** Chordomas at the Region S_3_ or below S_3_ accounting for 45.9% (145/316); **c** Chordomas associated with both high and low levels accounting for 52.5% (166/316); **d** GCT at the Region S_1_ or S_2_ or S_1-2_ accounting for 39.0% (80/205); **e** GCT at the Region S_3_ or below S_3_ accounting for 5.4% (11/205); **f** GCT associated with both high and low levels accounting for 55.6% (114/205)

To distinguish the high or low level was more important for the design of surgical strategy. Thus, we analyzed the location distribution of all tumors. For the malignant tumors, 412 were located at the region of above S_3_ and 304 tumors were at the S_3_/below S_3_. For the benign tumors, 485 were located at the region of above S_3_ and 184 tumors were at the S_3_/below S_3_. Chordoma and giant cell tumors are the top two histologic types of tumors at the sacrum. Among 316 cases with chordoma, only five (1.6%, 5/316) were located at Region S_1_ to S_2_, 145 (45.9%, 145/316) being at Region S_3_ and below S_3_ and 166 (52.5%, 166/316) were associated with both two regions. Meanwhile, among 205 cases with giant cell tumor, eighty tumors (39%, 80/205) were located at Region S_1_ to S_2_ and 114 (55.6%, 114/205) cases were associated with both two regions. Only eleven giant cell tumors were located at Region S_3_ and below S_3._ Notably, there was a significant difference in location distribution between chordoma and giant cell tumor (Table 7). We showed typical cases with chordoma and giant cell tumor in different regions of the sacrum (Fig. 5). Chordoma usually originated at the low level of the sacrum and extended upward and forward. Giant cell tumor often stemmed from the high level of the sacrum and destroyed the lower level. This characteristic was more useful to distinguish chordoma from the giant cell tumor.

**Table 7:** Comparision of location distribution of top two histologic types

Location	Chordoma (***n*** = 316)	Giant cell tumor (***n*** = 205)	***P*** value
Region S_1_ or S_2_ or S_1-2_	5 (1.6%)	80 (39.0%)	<0.001
Region S_3_ and below S_3_	145 (45.9%)	11 (5.4%)	
Associated with both two regions	166 (52.5%)	114 (55.6%)

## Discussion

This series of primary sacral tumors and tumor-like lesions treated at our tumor center provides valuable data of clinical characteristics contributing to our understanding of the diagnosis and therapy for lesions at the sacrum in the clinical practice. Available reports on clinical features of primary spine tumors and tumor-like lesions were mostly among the mobile spine [1, 4, 5]. There are not enough studies to reveal the clinical characteristics of patients with sacral tumor and tumor-like lesions in China. In the literature review, Zhou et al. reported 68 benign and 134 malignant sacral tumors in the analysis of clinical features for spine tumors. In their study, giant cell tumor, hemangioma, and solitary bone cyst were the top three benign tumors meanwhile chordoma, malignant neurilemmoma, and chondrosarcoma were the top three malignant tumors. The incidence characteristic of their malignant tumors was basically similar to our cohort. Nevertheless, the rate of their benign tumors was distinct from ours. This may be related to their limited sacral case numbers. The present report demonstrated clinical characteristics of age, gender, histologic type, and anatomic site based on the largest series of sacral tumors and tumor-like lesions.

Clinical characteristics of patients with sacral tumor and tumor-like lesions could help us to perform the differential diagnosis. Chordoma, chondrosarcomas, myeloma, and lymphoma more frequently occurs at the older age, otherwise, osteosarcoma and Ewing sarcoma are more frequent at a young age. Chordoma and teratoma are commonly located at the lower level. For malignant tumors, osteosarcoma, myeloma, and chordoma at the sacrum have male predilection while MPNST is more frequent in female population. For benign tumors and tumor-like lesions, epidermoid cyst and teratoma have the obvious female predilection.

In our series, chordoma was the most common primary malignant tumor, accounting for 44.1% (316/716), followed by chondrosarcoma (10.3%, 74/716), myeloma (7.4%, 53/716), malignant peripheral nerve sheath tumor (6.6%, 47/716), Ewing sarcoma (6.6%, 47/716), osteosarcoma (5.6%, 40/716), and lymphoma (4.9%, 35/716). Chordoma is a relatively rare neoplasm and accounts for 1–4% of all primary malignant bone tumors which arise from embryonic remnants of notochord [6]. It has been reported that chordomas occur most commonly within the sacrum (50–60%), followed by the spheno-occipital vertebrae (25–30%), cervical region (10%), and thoracolumbar vertebrae (5%) and chordoma affects males more commonly than female [7]. Our results also showed the male predominance was most pronounced among 306 chordomas, accounting for 68.6% and the age of 51–70 yrs was the most common of age interval, which was concordant with the previous studies. The peak age in our cohort was the range of 51 to 60 and it was distinctly uncommon in patients younger than 30 years, only accounting for 5.9% (18/306). Furthermore, the peak age of chordoma was higher than that of chondrosarcomas, MPNST, and lymphoma.

Chondrosarcomas (CS) rank the second in the incidence of all primary malignant bone tumors, with reporting about 25% incidence and following after osteosarcoma. Regarding age, it is more common in adults between 40 and 80 years old and is slightly more common in men [8]. CS constituted over 20.4% of the malignant tumors in Mayo Clinic series and more than two-thirds of chondrosarcomas were in the trunk and the upper ends of the femur and humerus. CS was relatively rare and the incidence at the sacrum was 1.9% (24/1293) among all chondrosarcomas in the Mayo Clinic series [9]. However, in our cohort, the analysis of the clinical feature of CS showed an obvious predilection of male (M: F = 1.24:1) and the peak age ranged from 41 to 50 years. Moreover, chondrosarcoma was the second common histological type at the region of the sacrum for the primary sacral malignant tumors and the incidence of CS among the malignant tumors was 10.3% (74/716).

Although myeloma and lymphoma are not the primary sacral tumors, they are more common lesions at the sacrum in the clinical practice. It is necessary to analyze their clinical features to distinguish them from primary malignant sacral tumors. Myeloma, a tumor of hematopoietic derivation, is the most common primary neoplasm of the bone. There were more than 5000 patients with myeloma documented in the Mayo Clinic files. They reported 67.7% were males and the largest concentration of age was in the sixth and seventh decades of life. The well-known rarity of myeloma in patients who were younger than 40 years was shown in the Mayo Clinic series [9]. Likewise, myeloma at the sacrum had the gender predilection (M: F = 2.12:1) and 67.9% of all myelomas were males in our series. The median age of myeloma was 54.1 ± 13.2 yrs and the peak age ranged from 41 to 50 years. Only two patients in our cohort were younger than 30 years and no patient was in the first and second decades of life. Generally, the clinical characteristics of sacral myeloma were similar to other bony myeloma. Bone lymphoma is a rare disease. It is estimated that bone lymphoma is accounting for about 5% of extranodal lymphomas and 3–7% of all malignant bone tumors [10]. Parker et al. first described the malignant lymphoma of bone and separated it from Ewing sarcoma [11]. The Mayo Clinic database showed the 905 cases of malignant lymphoma comprised of 12.7% of the malignant bone tumors in their series and males predominated at a ratio of 4 to 3 in their cohort. In our cohort, approximately 51.4% of lymphoma occurred in patients between 41 and 60 years, with a peak incidence in the fifth and sixth decade of life. Only 5.7% of the patients with lymphoma were younger than 20 years and no one was younger than 10 years old. However, our series revealed lymphoma at the sacrum had the female predilection (M: F = 0.75:1), which was not concordant with the Mayo Clinic experience.

Malignant peripheral nerve sheath tumors (MPNST) include malignant schwannoma (malignant peripheral schwannoma) and neurofibrosarcoma. MPNST represents a relatively common subtype of soft tissue sarcoma and is particularly likely to occur in individuals with type 1 neurofibromatosis (NF1) [12–15]. A SEER database analysis showed 64 MPNST in the spinal location. Their mean age at diagnosis was 50.9 years with more patients in the higher age group and 56% of patients were male. However, it had no description of the incidence at the sacrum [16]. In our cohort, the analysis of the clinical feature of MPNST showed an obvious predilection of female (M: F = 0.68:1) and the peak age ranged from 41 to 50 years. Our result showed the clinical characteristic of age predilection was in concordance with the previous study in the SEER database, but gender predilection of MPNST at the sacrum was not similar to the result of the SEER database [16].

Ewing sarcoma (ES) and osteosarcoma (OS) are more frequent for young patients. The incidence of ES is a little higher than that of OS at the sacrum in the present study. ES is a distinctive, small, round cell sarcoma that is considered one of the most lethal of all bone tumors. In the Mayo Clinic database, ES comprised 8.6% of the total malignant tumors and had a distinct predilection for males (62%). Furthermore, approximately 75% were in the first two decades of life and the incidence of ES at the sacrum accounted for 5.9% (36/611) of all patients with ES [9]. The sacrum is not the frequent site for Ewing sarcoma. In our PKUPH database, Ewing sarcoma is located at the sixth top of all sacral malignant tumors. The peak incidence was in the second decade (40.4% of all 47 Ewing sarcomas), followed by the third and fourth decades (38.2%) and it had no predilection of gender, which was not concordant with the Mayo Clinic experience. Osteosarcoma is the most common malignant bone tumor. Mayo Clinic series files recorded 1952 osteosarcomas, accounting for 27.5% of all malignant tumors and 19.2% of all bone tumors and approximately 58% of patients with OS were male. In their database, the incidence of OS at the sacrum accounted for 2.0% of all patients with OS and the anatomic site around the knee was the most common site. The second decade was the most common age distribution and among 1952 osteosarcomas, 192 patients were older than 60 years who had pre-existing condition such as Paget disease, previous radiation, infarct, chronic osteomyelitis, and cyst of degenerative joint disease as the second peak [9]. According to our results, osteosarcoma at the sacrum was not frequent as the site around the knee and placed as the sixth top of all primary sacral malignant tumors. Although only one patient with the sacral OS was in the first decade of life, the peak incidence was in the second decade (37.5% of all 40 osteosarcomas), followed by the third decade (32.5%). We noticed no one patient aged more than 60 years. This result demonstrated that the age distribution of sacral osteosarcoma had the peak age of 11–20 and did not show another age peak of more than 60 years. Moreover, osteosarcoma was not common in the region of the sacrum. Thus, when an adolescent patient presents with a malignant tumor, we should take these two histologic types into consideration.

Our PKUPH database showed that giant cell tumor (30.6%, 205/669) was the most frequent in the cohort of benign tumors and tumor-like lesions, followed by neurofibroma (23.2%, 155/669) and schwannoma (17.8%, 119/669). The clinical characteristics of primary sacral benign tumors were distinct from the one of spinal benign tumors. Some rare histologic types should be considered when the differential diagnosis [17–23]. It has been reported that hemangioma was the most frequent benign primary spine tumor in one hospital of China, which was different from the region of the sacrum and our database showed giant cell tumor was the most common histological type at the sacrum [1]. Hemangioma at the sacrum accounted for 2.1% (14/669) in the cohort of benign tumor and tumor-like lesions. It illustrated that hemangioma was not a frequent tumor at the sacrum, which was different from the characteristics of the mobile spine. It is more important to distinguish some benign histologic types such as giant cell tumor, hemangioma, and aneurysmal bone cyst, due to their higher vascularity and intraoperative hemorrhage. Thus, although these lesions are benign, more attention should be paid to the problem of intraoperative blood loss.

Giant cell tumor (GCT) is an invasive benign bone tumor consisting of proliferative mononuclear cells and osteoclast-like multinucleated giant cells. In the present study, GCT was the most common benign tumor at the sacrum. This result was concordant with the study of Zhou et al. about the epidemiological feature of all spinal tumors [1]. The Mayo Clinic series showed the female predominated in bone GCT, with 376 females and 295 males [9]. However, Niu et al. reported 621 patients with GCT in the extremity and male predominance (1.4:1) was observed in their series [24]. Their epidemiological feature of extremities was different from the characteristics of our sacral GCT and this illustrated that predominate gender may depend on the anatomical site. Our cohort also had 110 cases of females, accounting for 53.7% of all GCTs in our series. Approximately 79.5% of GCT occurred in patients between 21 and 50 years, with a peak incidence in the third and fourth decade of life. Only 8.8% of the patients with GCT were older than 50 years and only 11.7% of GCT occurred in patients before 20 years old. Furthermore, no one was younger than 10 years old. Thus, it illustrated that giant cell tumors are not common in the adolescent population.

Benign peripheral neurogenic tumors include neurofibroma and peripheral schwannoma. Neurogenic tumors arising from the sacrum are rare, with only about 7% of intraspinal neurogenic tumors involving the sacrum. Neurofibromas can occur in any site, both deep soft tissue and superficial cutaneous lesions. They are usually painless and they are often excised for cosmetic purposes. Individuals with NF1 may have multiple neurofibromas that need excision for functional purpose as well as to exclude the possibility of malignant transformation. The analysis of the clinical feature of neurofibroma in our series showed a little predilection of females (M: F = 0.80:1) and the peak age ranged from 41 to 60 years. Meanwhile, schwannomas are relatively common benign lesions of the peripheral nerves, which are thought to derive from Schwann cell. They affect all age groups and are usually solitary sporadic tumors. Schwannomas can also develop along the spine adjacent to the neural foramina and frequently show a “target sign” on imaging that can be very suggestive of this specific diagnosis. They are usually slow-growing and often discovered incidentally. It has been reported that sacral schwannomas typically occur in middle-aged patients without any predilection for gender. Pennington et al. performed a systematic review and reported that pre-sacral schwannoma can reasonably be treated with either en bloc or piecemeal excision and recurrence was infrequent. In their review, the patients showed no gender predilection [25]. Our database analysis revealed the clinical feature of schwannoma also had no gender predilection and the peak age ranged from 31 to 60 years. This result was concordant with the result of the previous study in the literature. Neurofibroma and schwannoma are always located at a higher level of the sacrum, which is distinct from the teratoma and we can consider this feature when the differential diagnosis is performed.

There were several limitations to the present study. Firstly, although the present cohort in our tumor center is large, the data drawn from the present analysis may not necessarily reflect the epidemiologic characteristics of the whole nation. Secondly, the present data lacked the information on the prognosis for all patients. Thus, in the next step, we will collect more information about the oncological prognosis of all patients.

In conclusion, the large cohort of sacral tumors and tumor-like lesions in our database may reveal their clinical characteristics in China and clinical feature of age, gender, histologic type, and anatomic site for sacral tumors and tumor-like lesions is fairly distinct from the mobile spine and extremities. 

## Data Availability

All data generated or analyzed during this study was included in this published article.
